# Predicting no-shows for dental appointments

**DOI:** 10.7717/peerj-cs.1147

**Published:** 2022-11-09

**Authors:** Yazeed Alabdulkarim, Mohammed Almukaynizi, Abdulmajeed Alameer, Bassil Makanati, Riyadh Althumairy, Abdulaziz Almaslukh

**Affiliations:** 1 Information Systems Department, King Saud University, Riyadh, Saudi Arabia; 2 Computer Science Department, King Saud University, Riyadh, Saudi Arabia; 3 Department of Restorative Dental Sciences, King Saud University, Riyadh, Saudi Arabia

**Keywords:** Dental no-shows, Machine learning, Patient no-show, Dental appointments

## Abstract

Patient no-shows is a significant problem in healthcare, reaching up to 80% of booked appointments and costing billions of dollars. Predicting no-shows for individual patients empowers clinics to implement better mitigation strategies. Patients’ no-show behavior varies across health clinics and the types of appointments, calling for fine-grained studies to uncover these variations in no-show patterns. This article focuses on dental appointments because they are notably longer than regular medical appointments due to the complexity of dental procedures. We leverage machine learning techniques to develop predictive models for dental no-shows, with the best model achieving an Area Under the Curve (AUC) of 0.718 and an F1 score of 66.5%. Additionally, we propose and evaluate a novel method to represent no-show history as a binary sequence of events, enabling the predictive models to learn the associated future no-show behavior with these patterns. We discuss the utility of no-show predictions to improve the scheduling of dental appointments, such as reallocating appointments and reducing their duration.

## Introduction

Patient no-show, which refers to patients missing their booked appointments, is a major problem in healthcare. Its rates may reach up to 80% (Kheirkhah et al., 2015; Rust et al., 1995). Reports estimated that the no-show of patients costs the United States’ healthcare system an average of $150 billion a year (Gier, 2017). Patients may miss their appointments for various reasons, such as scheduling factors, forgetness, transportation, and work-related issues (Marbouh et al., 2020). The consequences of this problem are wide, affecting patients, doctors, and the overall healthcare system. Patients are perhaps affected the most by no-shows, whether it is the patient who has missed the appointment or the others who could not book appointments due to unavailabilities. When patients miss their appointments, important treatments get delayed, leading to complications that require costly and complex medical procedures.

Outpatient clinics implement several mitigation strategies to tackle the no-show problem, yet, this brings limited success. For example, sending reminders to booked patients may not reduce the no-show rates (Li et al., 2019b). Blind overbooking is ineffective because the number of extra appointments is often estimated based on factors that do not consider the historical no-show patterns of the scheduled patients. If the overbooking strategy is implemented aggressively, it would crowd the clinic, over-utilize its resources and increase patient waiting time. On the other hand, if overbooking is conservative, resources would be wasted, and the clinic is yet again underbooked (Zeng, Zhao & Lawley, 2013).

Several papers in the literature have utilized machine learning techniques to develop predictive models for patient no-shows. See “Related Work” for details. These models use patient no-show history to predict no-shows on a per-patient basis, which empowers targeted and more effective mitigation strategies. No-show behavior varies across health clinics and specialties (AlMuhaideb et al., 2019; Ding et al., 2018; Mohammadi et al., 2018). For instance, reported percentages of no-shows for Urology and Oncology were 6.5% and 6.9%, respectively. In contrast, no-shows for Surgery, Optometry, and Dietary reached 16.6%, 23.8% and 27.23%, respectively (AlMuhaideb et al., 2019). This calls for fine-grained studies covering specific specialties to discover useful patterns that may help to predict no-shows.

This problem could get even worse for some geographical locations. For example, previous research reported that 58.1% of patients missed their dental appointments in Saudi Arabia, impacting their oral health (Baskaradoss, 2016; Shabbir, Alzahrani & Khalid, 2018). Furthermore, dental appointments are notably longer than regular medical appointments due to the complexity of dental procedures. The average duration of primary care appointments is 17.4 min, while the average for dental appointments is 48.7 min, as reported by the American Dental Association (American Dental Association, 2021). Dental appointments are, on average, almost thrice as much as the duration of primary care appointments. Consequently, dental no-shows are costlier and blind overbookings are impractical. In this article, we leverage machine learning techniques on a newly proposed feature creation approach to develop models to precisely predict no-shows to dental appointments.

Prior no-show history is one of the most significant predictors for future no-shows (AlMuhaideb et al., 2019; Goffman et al., 2017; Harvey et al., 2017). This article proposes a novel method to represent no-show history as a binary sequence of events. Each previous appointment is represented as 1 if the patient has attended and as 0 if the patient has missed the appointment, resulting in a representation of a sequence of binary numbers for each of the appointment records. We evaluate this approach for capturing no-show history considering the last three, five, seven, and ten appointments. The intuition behind this approach is that the model should learn the behavior associated with each sequence of no-show history in the training phase rather than reducing no-show history to a number based on fixed calculations.

Additionally, we discuss the utility of no-show predictions to improve the scheduling of dental appointments. Several studies (Chiara & Domenic, 2018; Li et al., 2019b; Srinivas & Ravindran, 2018) suggested utilizing the predictions of no-shows to implement more effective overbooking strategies. These strategies may be impractical for dental appointments because of their long duration, see “Discussion” for details. For this reason, we propose to reduce the time of appointments with high no-show probabilities to minimize the impact of no-shows. Last, the dataset is publicly available for the research community to facilitate further research and comparative studies. The contribution of this article is as follows:

- Leveraging machine learning models to predict no-shows for dental appointments.
- Proposing a novel method to capture prior no-show history, one of the strongest predictors of no-shows, as a binary sequence.
- Reviewing and discussing various techniques to mitigate no-shows in the field of dentistry.
- Making the dataset publicly available for further research (see Supplemental File).

The organization of this article is as follows. “Problem Definition” details our approach to capturing no-show history. The collected data and our research methodology are described in “Data Analysis” and “Experimental Setup”, respectively. Experimental results and their discussion are reported in “Results” and “Discussion”. Last, “Related Work” provides a literature review and “Conclusion” concludes our work.

## Problem definition

In this section, we first formally define the problem of predicting no-shows to dental appointments. Then we describe our approach to capturing the no-show history patterns from our dataset.

### Predicting dental appointment no-shows

We formulate the problem of predicting no-shows as a binary classification problem, where the goal is to assign a class label belonging to {no-show, show} for each booked dental appointment. In our settings, we consider the no-show as the *positive class* for which our models are optimized to predict. For a given booked dental appointment $n_i$, such that $n_i \in N$, where *N* is the set of all booked appointments, we seek to define a prediction algorithm that assigns a score $S(n_i)$ $\in$ [0, 1]. The score models the predicted likelihood that the dental appointment $n_i$ will be unattended, *i.e.*, the higher the prediction score, the more likely that patient will not attend the appointment. A decision boundary $\gamma$ $\in$ [0, 1] can be used to attain class label assignments in accordance to the prediction function *F*: *N* $\rightarrow$ [no-show, show], such that:

$F(N) = \left\{ \matrix{
  {\rm{no - show}},\quad if\quad S(N) \ge \gamma , \hfill \cr 
  \quad {\rm{show}},\quad \quad {\rm{otherwise}}. \hfill \cr}  \right.$

### Capturing no-show history

Prior no-show history is one of the significant predictors for future no-shows (AlMuhaideb et al., 2019; Chiara & Domenic, 2018; Goffman et al., 2017; Harvey et al., 2017). Most papers in the literature capture it as an empirical probability, a likelihood of an event occurring based on historical data. Instead of computing the no-show history for all previous appointments, the no-show percentage is usually calculated based on a moving window, such as the latest 10 appointments. This is because older appointments have less impact on predicting future no-shows than recent ones.

This article captures no-show history as a binary sequence of events, representing each previous appointment as 1 if the patient attended and 0 if the patient missed the appointment. For instance, a patient with a previous no-show history sequence as (show, no-show, no-show, show, no-show) is represented as a (1, 0, 0, 1, 0). This approach is applied to sequences of $n$ last appointments, *i.e.*, $( a_i) _{i=1}^n, a_i \in \{0,1\}$. We perform an evaluation for these sequences where $n \in \{3,5,7,10\}$; see “Results” for details. The intuition behind this method is that the model should learn the behavior associated with each sequence of no-show history in the training phase rather than reducing no-show history to a single number, *e.g.*, moving average. For example, consider a patient with a no-show history sequence as (show, show, no-show, no-show). The empirical probability of this no-show history is 50%. This approach treats all previous appointments equally, computing the same percentage (50%) for other different sequences, such as (no-show, no-show, show, show). It fails to capture possibly different future no-show behavior associated with these sequences.

## Data analysis

In order to evaluate our approach, we collected and analyzed a dataset of dental appointments obtained from a dental clinic. In “Data Collection and Preprocessing ”, we describe our approach for collecting and preprocessing the dataset, and in “Feature Analysis ”, we describe our feature analysis process.

### Data collection and preprocessing

For this article, we collected a dataset of dental appointments from a local dental clinic in Riyadh, Saudi Arabia, for one year between January 1st, 2019, and December 31st, 2019. Our dataset consists of 262,140 appointments. Among these appointments, we delete 66,122 records that are missing critical data elements, such as appointment date/time, or containing erroneous values (see “Data Preprocessing” for details). The resulting data consists of 196,018 appointments. Among these appointments, 42.68% (83,663) belong to the positive class, *i.e., no-show*.

Each record in our dataset consists of 10 predictive attributes (features), in addition to the target class (*show* or *no-show*). Among the predictive attributes are data specific to each patient, such as date of birth, marital status, gender, and nationality. The records also contain information about the appointments, such as appointment date, time, *show* or *no-show*, booking date and time, appointment duration, and a variable specifying whether a confirmation SMS is sent for that appointment. Description of each variable is shown in Table 1.

**Table 1:** Description of the variables in the dataset.

Category	Variable	Description
	Date of birth	Patient’s date of birth
	Marital status	Specifies whether the patient is single or married
	Gender	Patient sex (male or female)
Patient	Nationality	The nationality of the patient
	Appointment date	Date of the booked appointment
	Appointment time	Time of the booked appointment
	Show or No-show	Specifies whether the appointment is attended or missed
	Booking date and time	Date and time in which the appointment was booked
	Appointment duration	Length of the appointment in minutes
	SMS	Specifies whether a confirmation SMS is sent or not sent
Appointment	Doctor ID	Dentist identification number

### Feature analysis

**Figure 1:**
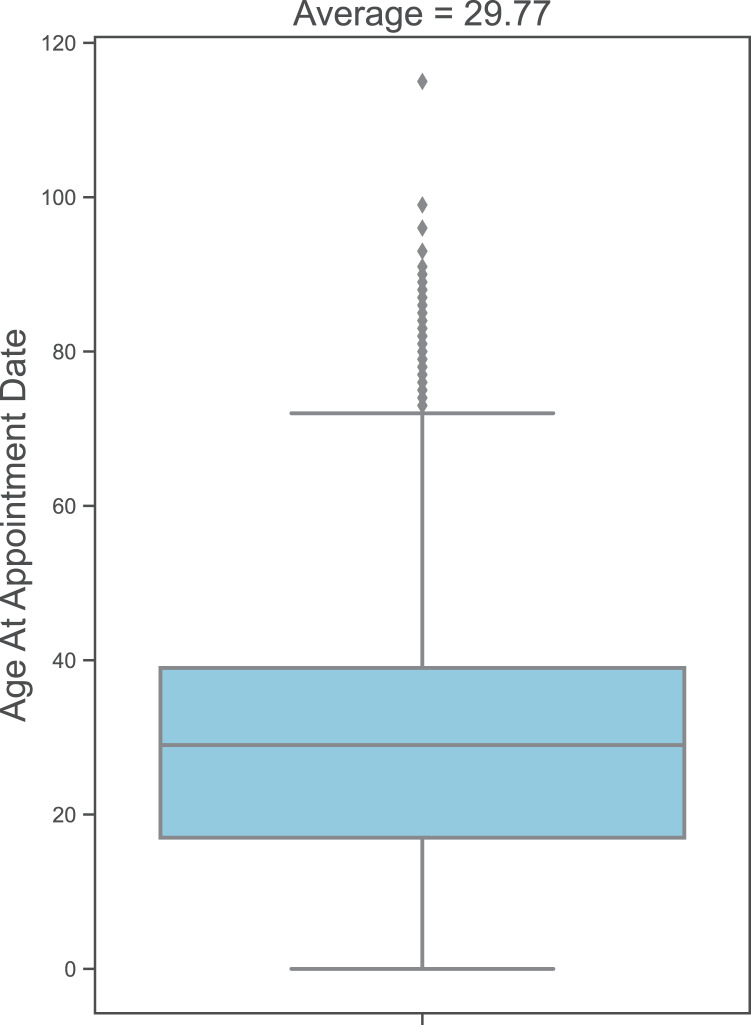
Patients’ age distribution at appointment date.

**Figure 2:**
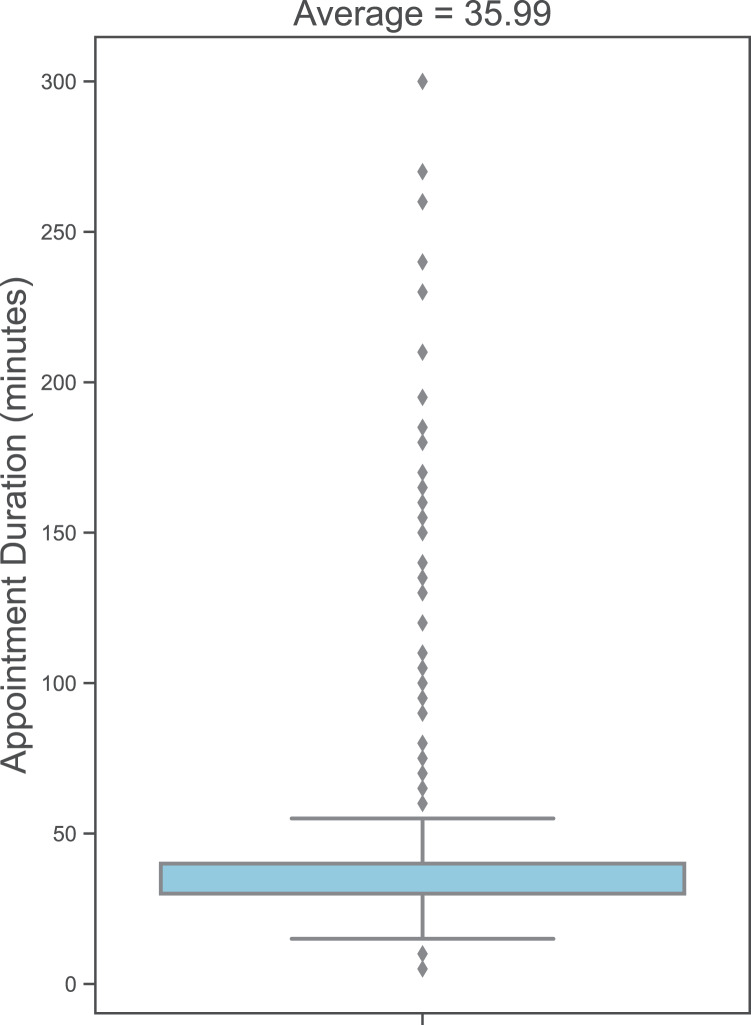
Distribution of appointment duration in minutes.

We explore the dataset to determine the distribution of values for different variables. In our dataset, 75.4% (133,772) of the appointments are for male patients, while 24.6% are for female patients; 89.3% are for single patients, and 10.7% are for married patients. The average age of patients at appointment time is 29.77 years, as illustrated in Fig. 1. The average appointment duration is 35.99 min, as shown in Fig. 2.

We utilize the variables, shown in Table 1, to compute features, such as the appointment lead time (*i.e.*, days between appointment booking date and actual appointment date), appointment month, and day. Furthermore, we obtain historical weather data for the appointment date, including temperature and condition. The complete list of computed features and their description is shown in Table 2. For instance, (*In Holiday*) is a feature to capture whether the appointment date is on a public holiday in Saudi Arabia, and (*In Ramadan*) is a feature to capture whether the appointment date is in Ramadan, which is the holy month of fasting for Muslims. We also compute features to capture the previous no-show history in various ways. First, we calculate the percentage of no-shows based on the last 10 appointments, namely ‘No-show10 %’, and based on all previous appointments, namely ‘No-show All %’. Second, we represent previous no-show history as a binary sequence of events. We provide a detailed description of the no-show binary sequence representation in “Problem Definition”.

**Table 2:** Description of the computed variables in the dataset.

Variable	Description
Multi appointments	More than one appointment on the same day
Age at appointment date	Patient age at the appointment date
Lead time	Days between booking and appointment dates
Days since last appointment	Days between the last and upcoming appointments
Appointment day	Day of the week that the appointment is booked in
Is weekend	If the appointment is on the weekend
Month	The month that the appointment was booked in
In holiday	If the appointment is scheduled to be during a national holiday
In Ramadan	If the appointment is in Ramadan
Temperature	The temperature on the appointment day
Weather	The weather on the appointment day
Hour	The appointment time in hours
No-show All #	Number of all no-shows
No-show All %	Percentage of all no-shows of a particular patient
No-show10 #	Number of no-shows based on the last ten appointments
No-show10 %	Percentage of no-shows based on the last ten appointments
No-show Seq3	Binary sequence representation of last three appointments
No-show Seq5	Binary sequence representation of last five appointments
No-show Seq7	Binary sequence representation of last seven appointments
No-show Seq10	Binary sequence representation of last ten appointments

We investigate the correlation between the target variable (*show* or *no-show*) and each feature in the dataset for preliminary analysis. We observe multiple features that correlate with the target class variable. These features are Age at Appointment Date, Lead Time, Hour, and the variables associated with previous no-show history.

**Figure 3:**
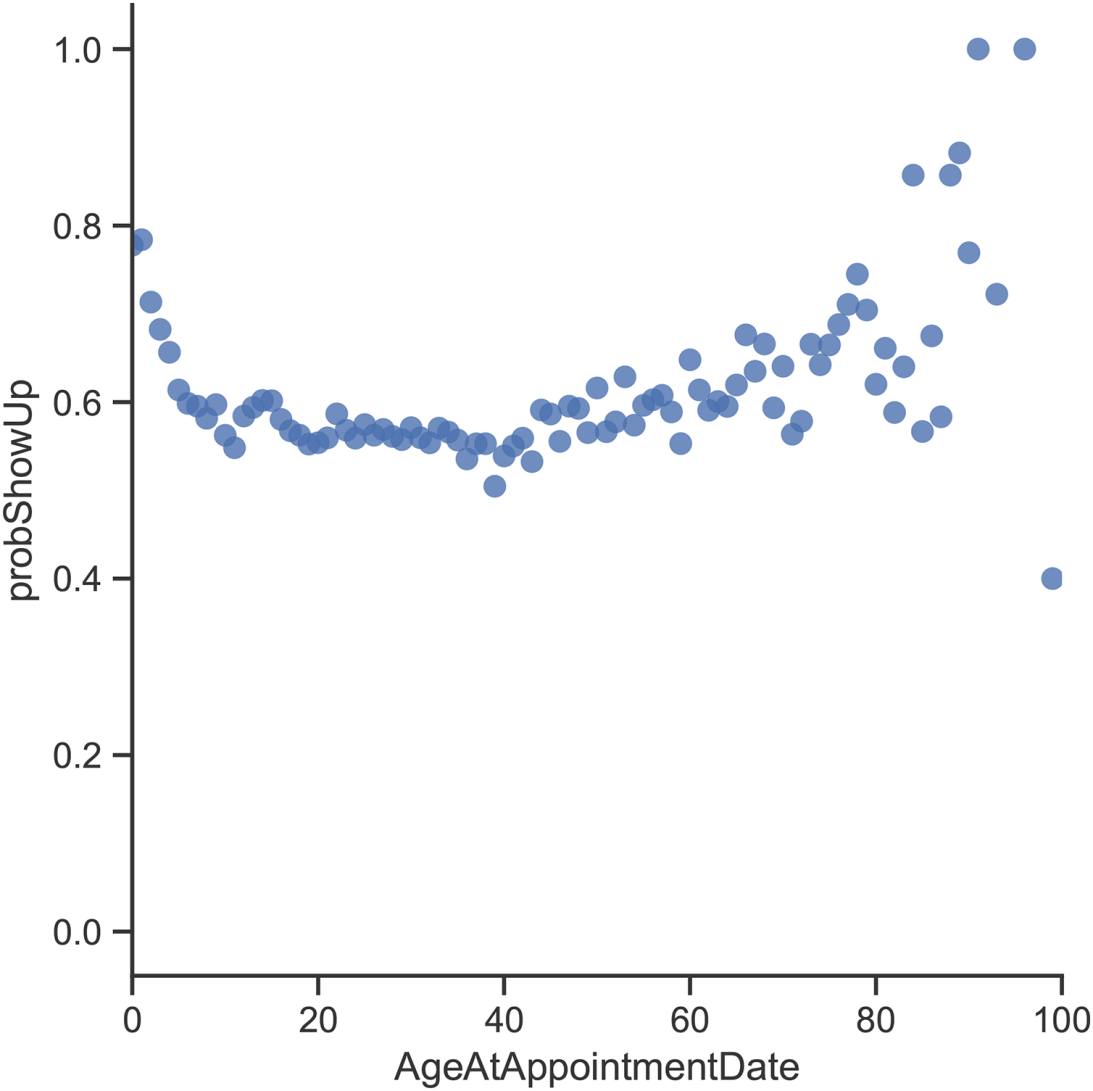
Probability of showing up with respect to the patient’s age at appointment date.

**Figure 4:**
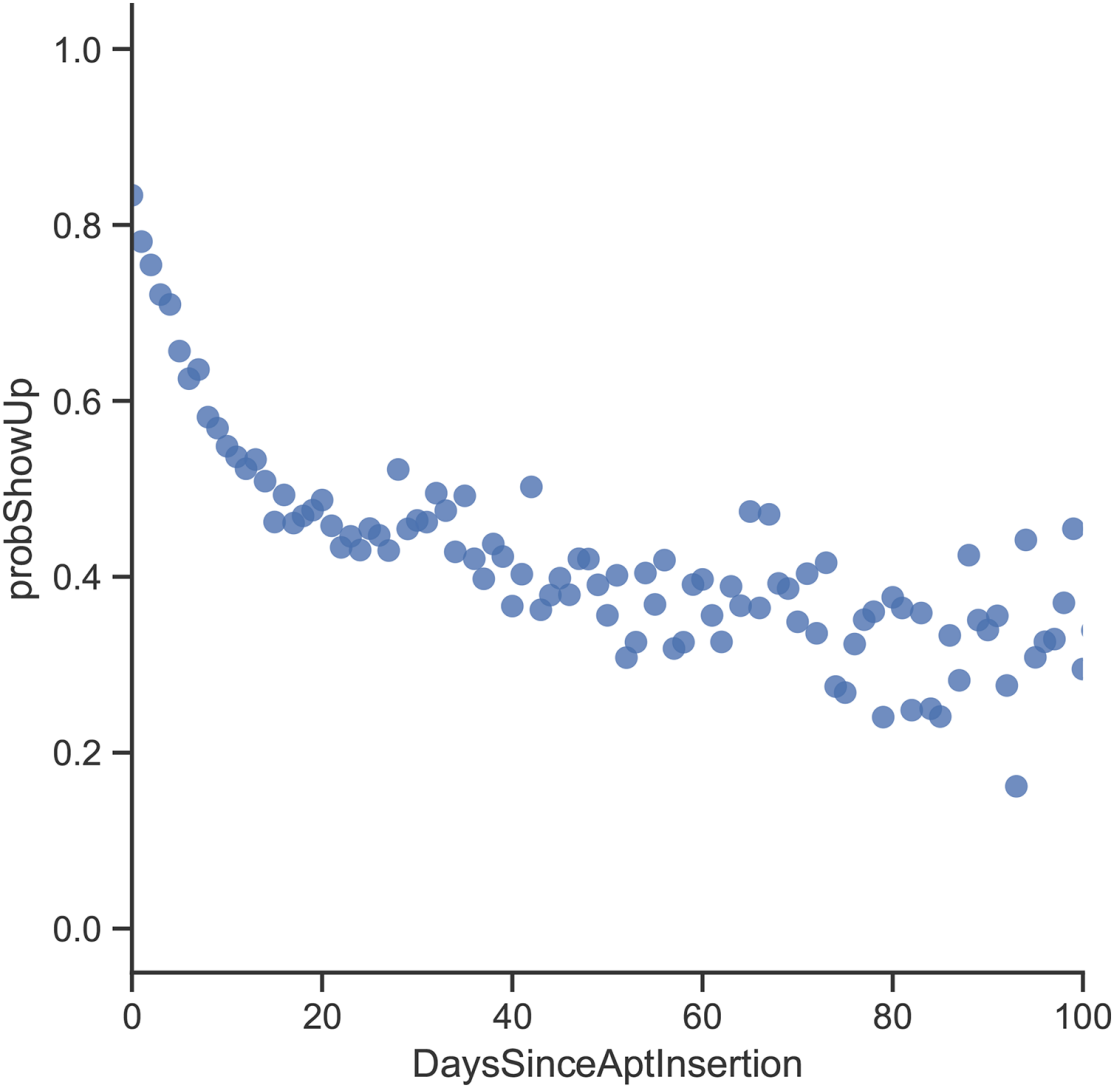
Probability of showing up with respect to lead time.

**Figure 5:**
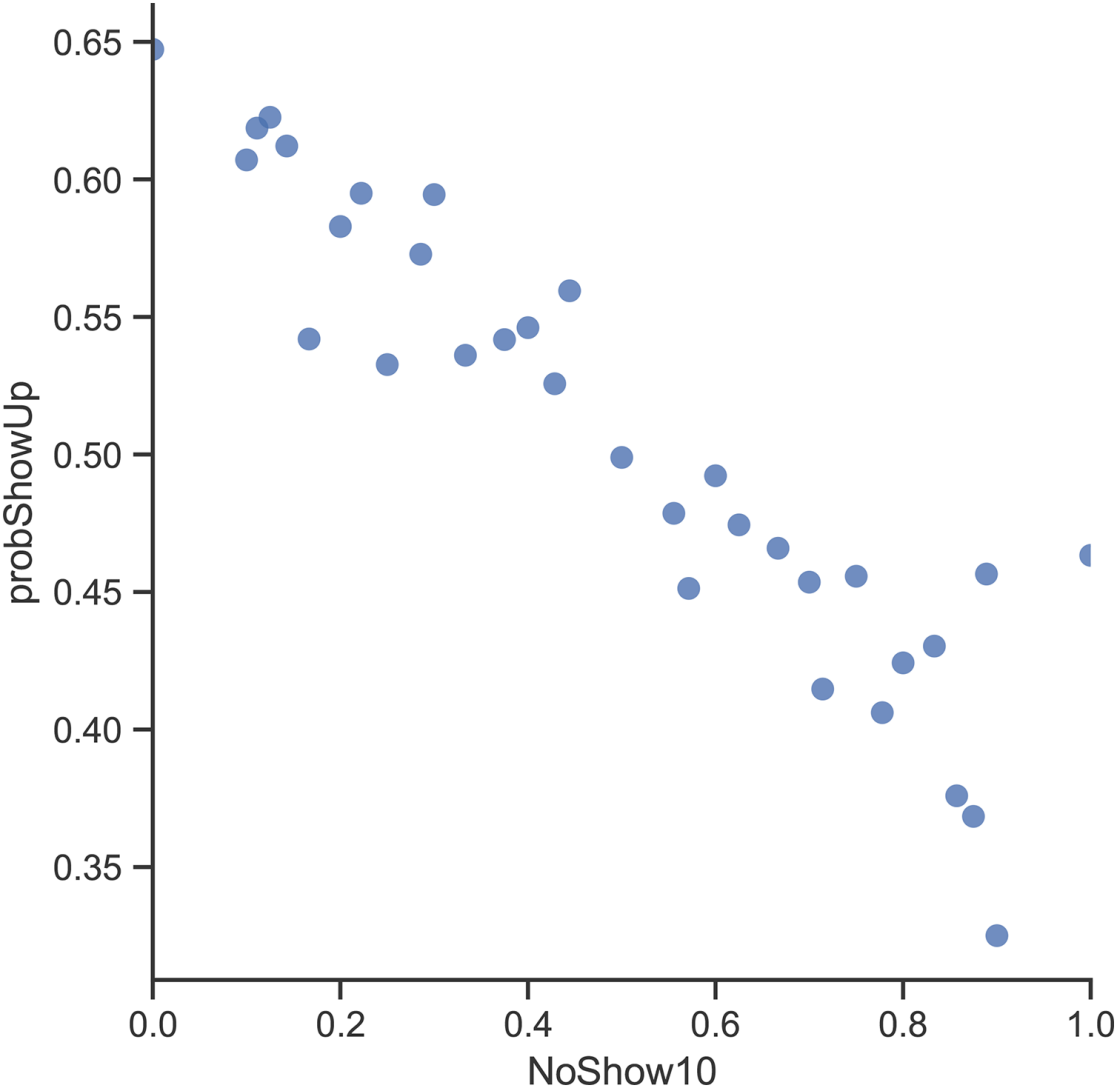
Probability of showing up with respect to no-show rate in the last 10 appointments.

By looking at the age variable (Fig. 3), the records in the dataset indicate that patients who need a companion (*i.e.*, kids and elderly patients) are more likely to show up for their appointments. By investigating the Lead Time variable (Fig. 4), it can be clearly seen that appointments that are booked closer to the actual appointment date have a higher rate of showing up. When looking at previous no-show history (Fig. 5), it is evident that patients with a high rate of prior no-shows are more likely to not show up for their future appointments.

**Figure 6:**
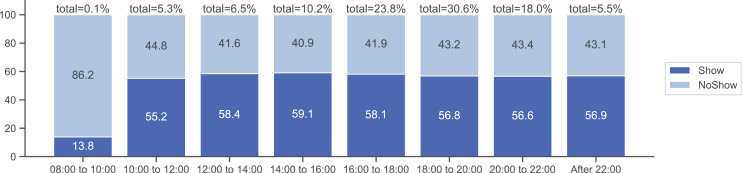
Probability of showing up with respect to the hour shift of the day.

To further investigate the relationship between appointment hours and the likelihood of no-shows, we bin the ‘Hour’ variable into different shifts to identify shifts that are likely to exhibit more no-shows. As seen in Fig. 6, appointments scheduled early in the morning (*i.e.*, before 10 AM) have the highest likelihood of being unattended.

Overall, this preliminary analysis shows clear correlations between some features and the target class label. This implies that machine learning methods can be effective in building a well-performing model for predicting no-shows for dental appointments.

## Experimental setup

Several supervised learning algorithms are examined to predict appointment no-shows with the best performance possible. This section explains the steps followed to preprocess and clean our data. It details our approach to partitioning data into training and testing sets. Moreover, the model selection process and hyperparameter optimization applied to our datasets are explained in this section. Additionally, we present the evaluation metrics used to evaluate the performance of the machine learning models. We do so by using the scikit-learn Python machine learning library.

### Data preprocessing

Our data is produced by an appointment scheduling system that enables the dental clinic to manage patient appointments. The data is pulled out from a relational database. Some attributes in the database may have been manually altered by users having certain privileges without validating the inputs. Manual alteration has resulted in issues with values in some database attributes. For example, the attribute ‘duration’ represents the estimated time needed to complete the dental appointment’s work. Hence, it may only take positive values. The dataset has zero- and negative-duration appointments. All records with any such invalid values have been removed from the dataset. Invalid values are found in four attributes, explained in Table 1. That includes records where: (1) ‘Appointment Duration’ is less than 10 min, (2) ‘Date of Birth’ is a future date, (3) ‘Appointment Date’ is missing, or (4) ‘Appointment Time’ is missing. Records of patients without appointments (*i.e.*, walk-ins) are removed because they are only admitted if physicians are available. Therefore, they are not part of the prediction task.

### Training and testing data split

Machine learning models are generally trained by fitting a training dataset. They are then evaluated on a different testing dataset. The train and test datasets are often assumed to be drawn from the same underlying data distribution. However, this may not always be the case with time-series data where the data distribution changes over time, aka., concept drift (see (Gama et al., 2014) for a survey). Data may be partitioned into training and testing data in different ways. For example, in k-fold cross-validation, performance is reported on all the data after k runs, *i.e.*, data is partitioned into k folds. For each fold/run, models are fit to the remaining k−1 folds, and the fold is used to test the models. Such a setting violates the temporal aspect of time-series data where concept drift may exist.

Our task of predicting appointment no-show has the temporal dimension, *i.e.*, time-dependent. Therefore, we follow experimental settings that best resemble real-world, deployed predictive models on time-series data where concept drift may exist. To do so, appointments are sorted by the appointment date for all experiments. After sorting, 90% of the data points are used to train models, and 10% of the data points are reserved for testing the models. We do so to avoid timely-intermixing the appointments. This way guarantees that none of the appointments in the testing set preceded any appointment in the training set. Following these settings, the first set of experiments is done to predict the no-show on all appointments, regardless of the appointment duration. In this set of experiments, models are trained on several feature sets, as explained in “Results”.

In another set of experiments, the models are fit to predict longer appointments, *e.g.*, appointments for more lengthy procedures such as crown mounting and root canal. This category of appointments costs the clinic significantly more than shorter appointments as they rely on the high involvement of skilled physicians and at least two assistants.

### Optimizing hyperparameters

Most supervised machine learning algorithms can be controlled to learn representations from data through a set of parameters, often known as hyperparameters, *e.g.*, learning rates, number of predictors in ensemble machine learning algorithms, depth of the decision trees, types of kernels, *etc*. The optimal set of hyperparameters should be empirically identified from the training dataset. The goal of such optimization is to produce the best-performing models (in terms of the efficiency of the learning process and the accuracy of the models). Grid search is perhaps the most commonly used hyperparameter tuning algorithm because of its implementation simplicity (Bergstra & Bengio, 2012). Grid search attempts to exhaustively search through a pre-defined set of hyperparameter space of a given algorithm.

In this article, we run a grid search for all experiments and models. Table 3 summarizes the hyperparameter search space and the optimal set of parameters that resulted in the highest F1 score. Grid search runs are parallelized on a CPU computer equipped with an 8-core Intel Core i7 Processor.

**Table 3:** Description of the hyperparameter search space and the optimal set of parameters that resulted in the highest F1 score.

ML model	Hyperparameter	Description	Search values	Optimal values by grid search
LR	‘penalty’	Specifies the norm of penalty term	L1, L2	L2
	‘C’	A constant controlling the regularization strength	0.001, 0.01, 0.1, 1, 10	0.001
	‘max_iter’	Maximum number of iterations for the solvers to converge	100, 250, 500	500
RF	‘n_estimators’	Number of trees in the forest	100, 250, 500	100
	‘max_depth’	Maximum depth of trees in the forest	5, 8, 10, 30, 40, 50	10
	‘min_samples_split’	Minimum number of samples required to be at a leaf node	2, 5, 10, 20, 50, 70, 80, 90, 100	70
GB	‘learning_rate’	A constant controlling the contribution of each tree	0.001, 0.01, 0.1, 1	1
	‘n_estimators’	Number of boosting stages performed	50, 100, 200, 300, 400, 500, 550, 600	500
	‘max_depth’	Number of nodes in each individual tree	2, 3, 5, 8	2
	‘min_samples_leaf’	Minimum number of samples required to be at a leaf node	1, 5, 10	1

### Supervised learning

After reviewing the related literature and empirically testing over eight different machine learning models, including k-nearest neighbors, naive Bayes, and decision tree, among others, we use grid search to find the optimal hyperparameter values on the three best-performing supervised learning models: (1) logistic regression (LR), (2) random forests (RF), and (3) gradient boosting (GB).

**Logistic regression (LR):** is a statistical model that is widely used for classification applications in various scientific fields. In logistic regression, a logistic function is fit to data to model the uncertainty that a dependant variable belongs to a certain binary class label, a setting that is similar to the problem addressed in this study. However, there are various extensions to logistic regression for multinomial classification problems (El-Habil, 2012).

**Random forests (RF):** is an ensemble learning model that is commonly used for classification (Breiman, 2001). A random forest works by constructing a set of classification tree predictors; each tree is induced from a set of data points sampled independently and trained on a sampled set of features, depending on the selected method. A large body of work in the literature has proposed different approaches to training individual trees in the forest for varying predictive tasks. In general, a significant improvement in predictive accuracy is achieved when the ensemble combines votes from the individual tree predictions.

**Gradient boosting (GB):** is similar to random forests, and it is an ensemble supervised machine learning approach that uses a set of decision trees. However, gradient boosting is an iterative model that builds the trees in a stage-wise manner by adding a weak learner at each learning stage to optimize the current ensemble of learners, *i.e.*, boosting (Friedman, 2002).

### Performance evaluation

To report the prediction performance, we compare the labels predicted by our models with the actual labels from the ground truth testing dataset. We use different evaluation metrics to quantify the prediction performance. The first set of metrics is the commonly used precision, recall, and F1 score. In the problem of predicting dental appointment no-shows, precision is the fraction of appointments that are correctly predicted to be unattended from the total number of predicted appointments, while recall is the fraction of appointments that are correctly predicted to be unattended from the total unattended appointments. F1 score is the harmonic mean of precision and recall. Table 4 summarizes how each metric is computed.

**Table 4:** Evaluation metrics. TP, true positives; FP, false positives; FN, false negatives; TN, true negative.

Metric	Formula
Precision	$ {TP}/{TP + FP}$
Recall	$ {TP}/{TP + FN}$
F1	$2 * {precision * recall}/{precision + recall}$

Another metric we use is the Receiver Operating Characteristic (ROC) curve, a graphical chart that illustrates the performance of a binary predictor. At each confidence score threshold, ROC plots the true positive rate against the false positive rate. True positive rate (aka., sensitivity or recall) in this study is the fraction of the correctly predicted no-show appointments from all no-show appointments. Moreover, the false positive rate (aka., false alarm ratio) is the fraction of the appointments that are wrongly predicted as no-shows from all attended appointments. ROC only shows the performance graphically. Therefore, we use the area under the ROC curve (AUC) to quantify the performance of the predictors. A perfect predictor would have an AUC of 1, while a random predictor with no discrimination power would be expected to have an AUC that is close to 0.5. AUC is reported for all results in “Results”.

## Results

**Figure 7:**
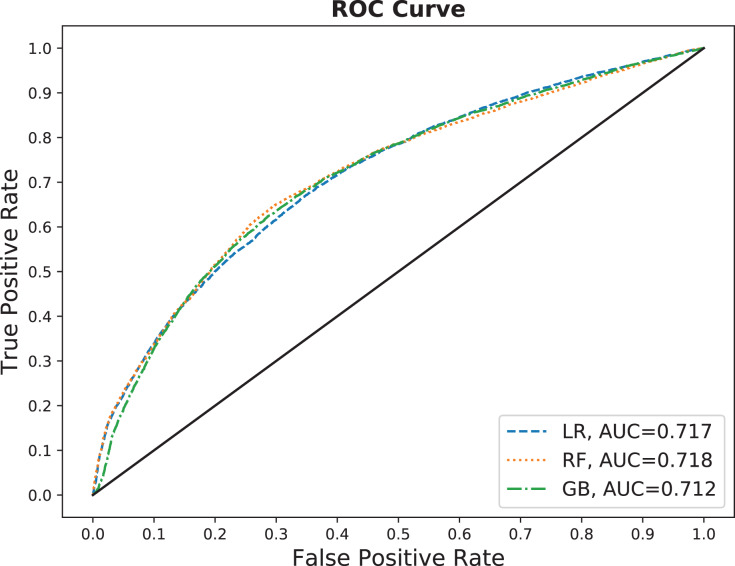
Receiver operating characteristic (ROC) curve comparing the three models on the testing dataset.

**Figure 8:**
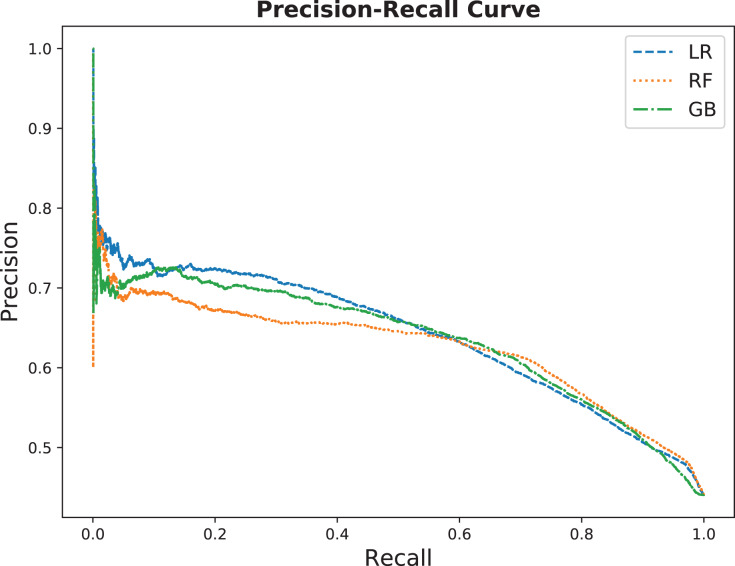
Precision-recall curve comparing the three models on the testing dataset.

This section shows the performance results for the three selected models explained in “Supervised Learning”. We choose ‘No-show Seq10’ of the no-show features, shown in Table 2, because it captures more history, see “Comparing No-Show Features” for details. Fig. 7 presents the ROC curve for the three no-show predictor models on the testing dataset. It is clearly shown that the three models are performing almost the same in terms of AUC. Yet, they are offering different tradeoffs between true positive and false positive rates. Such tradeoffs are presented in the precision-recall curve as in Fig. 8.

**Figure 9:**
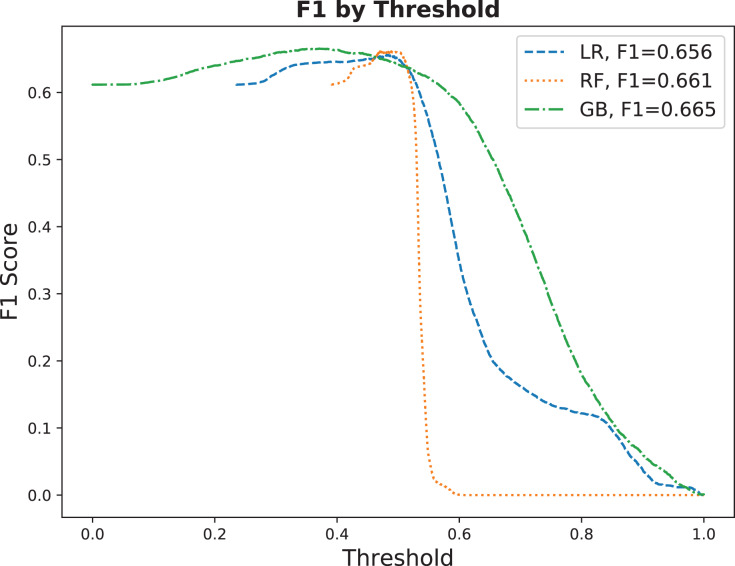
F1 by threshold comparing the three models on the testing dataset.

Next, we show the F1 scores and compare the three models across various threshold values in Fig. 9. The models have similar F1 scores, but it is noticeable that GB tolerates threshold changes better than LR and RF. Given that the prior no-show probability is 42.68% in our testing dataset, our highest model with an F1 of 66.5% outperforms the baseline with an F1 increase of over 55%.

In the following subsections, we show results for four sets of experiments: (1) experiments providing a comparison between the different no-show features, (2) experiments evaluating the performance of our no-show prediction models on appointments with long duration, (3) experiments reporting the results of an ablation test to identify important features that are most indicative of class label, and (4) experiments evaluating the generalization of our models on a different dataset.

### Comparing no-show features

This article proposes a novel method to represent no-show history as a binary sequence of events, as discussed in “Comparing No-Show Features”. To show the utility of the no-show history representation approach, we evaluate this approach for the last $n$ previous appointments $(a_i)_{i=1}^n, a_i \in \{0,1\}$. This sequence representation is compared to other methods that represent previous no-show history as a numerical feature, namely ‘No-show All #’, ‘No-show All %’, ‘No-show10 #’, and ‘No-show10 %’, as described in Table 2, to evaluate the value of learned no-show sequences.

Table 5 provides a comparison between these no-show features. Each row shows the reported AUC for one of the prediction models. The second column, ‘Baseline’, represents the baseline result for each prediction model where all no-show history features are removed. We keep all other features that are not related to no-show history in all experiments. Each of the remaining columns represents adding its corresponding no-show history feature.

**Table 5:** Reported AUC results for the three models on the testing dataset using various representations of no-show history.

Model	Baseline	No-show All #	No-show All %	No-show10 #	No-show10 %	No-show Seq3	No-show Seq5	No-show Seq7	No-show Seq10	Markov model
LR	0.676	0.674	0.695	0.677	0.695	0.718	0.719	0.718	0.717	0.712
RF	0.726	0.723	0.725	0.726	0.726	0.728	0.731	0.727	0.718	0.716
GB	0.724	0.726	0.724	0.732	0.727	0.728	0.722	0.722	0.712	0.711

Considering the logistic regression model, the binary sequence representations of no-show history outperform the numerical features by 2–5%. There is no significant difference between the examined lengths of no-show sequences. For the numerical no-show history features, percentage values outperform counter values by about 2%. For the remaining RF and GB models, there are insignificant differences between the various no-show history features. The reported AUC baseline numbers (second column) for the RF and GB models are very close to the AUC numbers when adding the no-show history features.

We compare our binary sequence representation of no-show history with the Markov model approach to compute no-show probabilities based on the last 10 appointments, proposed in (Goffman et al., 2017). The AUC values for the Markov model, see the last column of Table 5, are very close to our binary sequence representation for the three machine learning models.

### Long appointments

Appointments that have longer duration are more costly as they occupy more time slots and probably include advanced medical procedures. Consequently, these appointments are more important for predicting no-shows accurately. Furthermore, we hypothesize that they have lower no-show rates as patients should be more considerate not to miss them. For this set of experiments, we look at appointments with duration $\geq d$ where $d \in \{15,30,40,45,50,60\}$. We report the no-show rate and the number of appointments in training and testing data sets for each group $d$ in Table 6. It turns out that the no-show rates are very similar for the various duration groups. We do not report the AUC for the various duration groups because they are comparable, indicating an insignificant impact.

**Table 6:** No-show percentage and the number of appointments in the training and testing data sets for each grouping of duration.

	Appointment length					
Stats	$\geq 15$	$\geq 30$	$\geq 40$	$\geq 45$	$\geq 50$	$\geq 60$
No-show %	42%	43%	44%	43%	43%	43%
Number of training appointments	173,537	143,899	61,155	42,522	36,152	31,036
Number of testing appointments	8,599	7,225	3,234	2,249	1,869	1,631

### Important features

We perform an ablation test to determine the important features. First, we add all features and measure AUC as a baseline, see second column of Table 7. Then, we remove each feature independently and report AUC to determine the importance of the feature. In other words, each experiment includes all features except for the tested feature.^1^ 1Since we are r es may minimize the impact of each other. We show the results for two features only, see Table 7, as other features are insignificant with less than 2% difference. Lead time is the most significant feature causing the AUC to drop 7.5%, 7.8%, and 7.9% with LR, RF, and GB, respectively, an observation that is confirmed by the existing literature (AlMuhaideb et al., 2019; Chiara & Domenic, 2018; Goffman et al., 2017; Harvey et al., 2017) that appointment lead time is one of the strong predictors of future no-shows. Eliminating no-show history features causes the AUC to drop by 4.1% with the LR model and is insignificant with the other models.

**Table 7:** Reported AUC results for the three models showing the most significant features.

Model	All features	Lead time	No-show history
LR	0.717	0.642	0.676
RF	0.718	0.640	0.726
GB	0.712	0.633	0.724

### Model generalization

It is critical for machine learning models not to be limited to their training and testing datasets. This ensures they are widely applicable and adapt to data changes without significant performance degradation. To evaluate the generalization of the trained models, we obtain a new dataset from a small dental clinic in Riyadh, Saudi Arabia. The dataset consists of 49,007 appointments with 41% no-shows.

We use this new dataset to test the three machine models trained on the first dataset, which is from a different clinic. We slightly modify the training dataset by removing the ‘SMS’ feature, which specifies whether an SMS confirmation message has been received because the new dataset does not have it. We also use the ‘No-show Seq3’ feature instead of ‘No-show Seq10’ due to the small size of the new dataset that generated insufficient no-show history.

Table 8 shows the AUC and F1 scores comparing the performance of the three models that are trained on 90% of the first dataset. The second column of Tables 8a and 8b shows the results for the 10% testing of the first dataset, while the third column shows the results for the whole new dataset. The results for both AUC and F1 scores for the new dataset are very similar to scores on the first dataset, showing that the three models generalize very well. Furthermore, given a 41% no-show for the new dataset as a baseline, our models outperform it by a percentage increase of over 62% in the F1 score.

**Table 8:** Evaluating the generalization of our models that are trained on the first dataset on a new dataset.

(a) AUC		
Model	First dataset	New dataset
LR	0.708	0.706
RF	0.731	0.748
GB	0.727	0.710

(b) F1 scores		
Model	First dataset	New dataset
LR	0.652	0.657
RF	0.673	0.665
GB	0.669	0.653

## Discussion

The patient no-show problem has a tremendous negative impact on dental clinics financially and operationally. Classical strategies to mitigate this problem include overbooking and sending a reminder to the patients. However, these strategies have limited success as they may not consider patients individually. In the literature, several works have adopted machine learning algorithms to develop a model to predict patients’ no-shows based on various features. This work utilizes machine learning algorithms to build a very effective predictive model of the no-show patient and improves the implemented classical strategies.

Previous studies considered the patient history of no-shows and the lead time as the essential features in building the no-show model. Other features such as age, appointment time, duration, distance from the clinic, whether a reminder has been sent, etc., are also considered. However, the importance of these features is relatively less than the history of no-shows and the lead time as they do not provide important information to the model. Similarly, this work focuses on these two features as the main features to develop the model. However, the history of no-shows is further categorized into a sequence of binary (0 for missed appointments and 1 for attended appointments). This representation allows the model to learn the behavior rather than depending on statistical calculations.

Implementing traditional strategies, including overbooking or sending reminders blindly to the patients, could unexpectedly increase the overall cost or overwhelm the dental clinic resources, eventually diminishing the quality of the provided services. The proposed no-show model may help implement a careful overbooking strategy by scheduling a reduced appointment time slot for patients with a high no-show prediction in contrast to the traditional scheduling where all patients have the same appointment time length. Thus, it would help to balance the cost of no-shows and the use of the clinic resources effectively.

Our results demonstrate the importance of considering the right representation and weighting the importance of one feature over others. More specifically, our results of the developed models utilizing the sequence of binary representation show that the accuracy score has improved noticeably as the best model achieves AUC of 0.718 beating the baseline approach with a percentage increase of over 62%. Additionally, the results of evaluation of the generalization of the model, using the develop models to a new dataset, show that the developed models are not limited to a specific dataset confirming the generality and ensuring the applicability with negligible performance degradation.

Several potential applications could utilize our model. For instance, deploying a recommender system on top of the predictive no-show model could even boost certainty and improve costs, resources, and patient satisfaction. For a given patient, the recommender system, with the aid of the no-show model, suggests the optimal time slot at which she is more likely to attend. For example, if a patient had missed some appointments in the past in the early morning, the recommender system may suggest an afternoon time slot. Additionally, the system could suggest a reduced time slot appointment for a patient with a high no-show risk; even if the patient attended, the inconvenience for the following appointments would be minimal. On the other hand, her absence would not considerably affect the utilization of the available resources.

Our predictive model has some limitations. First, some variables in the datasets are unavailable, such as home address and distance to the clinic, which could even help the model accuracy overall and reveal more meaningful insights. Second, external factors such as the daily weather or some significant events have not been considered. Including such factors could improve the model accuracy.

## Related work

There is an abundance of papers that studied the behavior of no-shows in various domains (Lawrence, Hong & Cherrier, 2003; Li et al., 2019a; Phumchusri & Maneesophon, 2014), such as healthcare, airlines, hotel, and restaurant reservations. In the healthcare domain, earlier work in the literature (Dove & Schneider, 1981; Oppenheim, Bergman & English, 1979; Shonick, 1977) studied the factors of no-show behavior and evaluated the effectiveness of various interventions, such as sending reminders and charging service fees, to mitigate no-shows (Garuda, Javalgi & Talluri, 1998). For predicting no-shows and the number of no-show patients, early work used statistics and data mining techniques, such as decision trees (Dove & Schneider, 1981) and association rules (Glowacka, Henry & May, 2009).

These techniques are used to discover patterns and properties of the data. They are unable to effectively capture and predict complex behavior, such as no-shows, that involve several factors. Furthermore, these techniques are not able to adapt to changing behavior and new data effectively. They also failed to implement an appropriately targeted intervention such as using text messages or reminder calls 24 h in advance to reduce no-shows.

The early 2010s have seen a proliferation of studies to predict no-shows for individual patients, using various machine learning and probabilistic modeling techniques. This includes employing one or more techniques, such as logistic regression (Alaeddini et al., 2011; Alshaya, McCarren & Al-Rasheed, 2019; Chiara & Domenic, 2018; Ding et al., 2018; Goffman et al., 2017; Harvey et al., 2017; Lenzi, Ben & Stein, 2019; Mohammadi et al., 2018; Nelson et al., 2019; Salazar et al., 2020; Srinivas & Ravindran, 2018), decision trees (Salazar et al., 2020), random forests (Alshaya, McCarren & Al-Rasheed, 2019; Ferro et al., 2020; Nelson et al., 2019; Salazar et al., 2020; Srinivas & Ravindran, 2018), neural networks (Ferro et al., 2020; Mohammadi et al., 2018; Srinivas & Ravindran, 2018), gradient boosting (Alshaya, McCarren & Al-Rasheed, 2019; Denney, Coyne & Rafiqi, 2019; Nelson et al., 2019; Srinivas & Ravindran, 2018), NaïveBayes (Mohammadi et al., 2018), Bayesian Belief Networks (Li et al., 2019b; Simsek et al., 2021; Topuz et al., 2018), Support Vector Machine (Alshaya, McCarren & Al-Rasheed, 2019; Nelson et al., 2019), Hoeffding trees (AlMuhaideb et al., 2019) and others (Alaeddini et al., 2011; Denney, Coyne & Rafiqi, 2019). Carreras-García et al. (2020) conduct a comprehensive systematic literature review analyzing and highlighting the limitations of the proposed predictive patient no-show models.

Some papers used different techniques to capture features (Goffman et al., 2017), select variables (Simsek et al., 2021; Topuz et al., 2018), and balance the classes of data (Alshaya, McCarren & Al-Rasheed, 2019; Nelson et al., 2019; Simsek et al., 2021). Techniques include class weights (Nelson et al., 2019), random undersampling (Nelson et al., 2019; Simsek et al., 2021), Synthetic Minority Oversampling Technique (SMOTE) (Nelson et al., 2019; Simsek et al., 2021), and elastic net (Topuz et al., 2018). To effectively capture no-show history, Goffman et al. (2017) capture patients’ no-show history as binary sequences and uses a Markov model to compute a probability for each observed sequence of no-shows in the dataset. First, the training dataset is processed to compute the no-show probability for each observed sequence of no-show history. Each probability is computed by considering possible next future outcomes for the corresponding no-show history sequence in the dataset. Our work is similar in that it represents no-show history as binary sequences. However, our approach maintains the no-show history sequences and tries to learn the behavior associated with different sequences from data training, including all features. Their work requires processing the dataset to compute probability tables for different lengths of sequences and reducing them to numbers based solely on observed no-show history in the dataset.

Similar to our findings, several papers (AlMuhaideb et al., 2019; Chiara & Domenic, 2018; Goffman et al., 2017; Harvey et al., 2017) reported that the features of previous no-show history and appointment lead time were strong predictors of future no-shows. Dantas et al. (2018) summaries in a condensed systematic review of 105 studies focused on no-shows in appointment scheduling revealing that socioeconomic status and history of no-shows have a huge impact on the patients to miss their appointments. The importance of other features, such as insurance (Harvey et al., 2017), multiple same-day appointment (Goffman et al., 2017), gender (Chiara & Domenic, 2018; Simsek et al., 2021), age (Chiara & Domenic, 2018; Simsek et al., 2021), care cost (Nelson et al., 2019), season (Li et al., 2019b), count of previous appointments (Nelson et al., 2019), online doctor rating (Fan et al., 2021), and utilizing a local weather data (Liu et al., 2022), varies across studies. This is because the no-show behavior varies by demographics, clinic specialties, and other factors. This calls for fine-grained studies covering specific specialties, clinics (Ding et al., 2018; Mohammadi et al., 2018) and demographics to discover useful patterns that may help to predict no-shows. For example, (Mohammadi et al., 2018) collected data from 10 facilities and observed differences in the strong predictors of no-shows across clinics and facilities. Ding et al. (2018) examined patient appointments across 14 specialties, not including dentistry, and 55 clinics. Their results showed that fine-grained data, which captures clinic and specialty details, is more likely to perform better.

Several research papers have contributed by studying data from different populations and specialties, see Table 9. These papers collected data from the United States (Alaeddini et al., 2011; Denney, Coyne & Rafiqi, 2019; Ding et al., 2018; Goffman et al., 2017; Harvey et al., 2017; Li et al., 2019b; Mohammadi et al., 2018; Srinivas & Ravindran, 2018; Topuz et al., 2018), United Kingdom (Nelson et al., 2019), Brazil (Alshaya, McCarren & Al-Rasheed, 2019; Lenzi, Ben & Stein, 2019; Salazar et al., 2020; Simsek et al., 2021), Italy (Chiara & Domenic, 2018), Columbia (Ferro et al., 2020), and Saudi Arabia (AlMuhaideb et al., 2019). Their data covered various clinics and specialties, such as primary care (Denney, Coyne & Rafiqi, 2019; Ferro et al., 2020; Lenzi, Ben & Stein, 2019; Simsek et al., 2021; Srinivas & Ravindran, 2018), community health centers (Mohammadi et al., 2018), veteran facilities (Alaeddini et al., 2011; Goffman et al., 2017), Radiology (Chiara & Domenic, 2018; Harvey et al., 2017; Nelson et al., 2019), Pediatrics (Topuz et al., 2018), and a mix of specialities (AlMuhaideb et al., 2019; Ding et al., 2018; Li et al., 2019b). Compared to previous studies, this article focuses on predicting no-shows for dental appointments in Saudi Arabia.

**Table 9:** Studied specialties and population in the literature.

Study	Speciality	Location
Salazar et al. (2020)	Unknown	Brazil
Ferro et al. (2020)	Primary care	Columbia
AlMuhaideb et al. (2019)	Various	Saudi Arabia
Alshaya, McCarren & Al-Rasheed (2019)	Unknown	Brazil
Denney, Coyne & Rafiqi (2019)	Primary care	United States
Nelson et al. (2019)	Radiology	United Kingdom
Lenzi, Ben & Stein (2019)	Primary care	Brazil
Li et al. (2019b)	Various	United States
Simsek et al. (2021)	Primary care	Brazil
Chiara & Domenic (2018)	Radiology	Italy
Srinivas & Ravindran (2018)	Primary care	United States
Ding et al. (2018)	Various	United States
Mohammadi et al. (2018)	Primary care (community health centers)	United States
Topuz et al. (2018)	Pediatric	United States
Goffman et al. (2017)	Primary care (veterans health centers)	United States
Harvey et al. (2017)	Radiology	United States
Alaeddini et al. (2011)	Primary care (veterans health centers)	United States

No-show predictions are utilized to improve the scheduling of appointments, resulting in better utilization of resources and satisfaction of patients. No-show predictions may be used to guide overbooking decisions (Chiara & Domenic, 2018; Li et al., 2019b; Srinivas & Ravindran, 2018), facilitate targetted interventions (Li et al., 2019b; Simsek et al., 2021) and reduce the cost of appointment reminders (Ferro et al., 2020; Nelson et al., 2019). For example, Srinivas & Ravindran (2018) proposed multiple overbooking and sequencing strategies for multi-stage appointments. Similarly, Chiara & Domenic (2018) presented an algorithm to compute the minutes eligible for overbooking.

Li et al. (2019b) developed two models: one to predict whether a patient would confirm her appointment or not and the second to compute the no-show probability. The first model clusters patients into three groups, which may be used for targeted interventions, and its output is fed to the second model, along with other features. The output of the second model is used to improve overbooking strategies with respect to several parameters, such as patient waiting cost, clinic overtime cost, and idle time cost. Nelson et al. (2019) proposed an equation to calculate the benefit of sending reminders to a particular set of patients compared to all patients.

## Conclusion

We study the patient no-show problem in the context of the dental appointment where more than 40% of the patients miss their appointments for various reasons. Consequently, it has a significant negative impact on the clinic resources and the patient’s oral health. Traditional solutions such as overbooking and sending a reminder have limited success and failed to address the problem effectively. In this work, we utilize state-of-the-art machine learning algorithms to develop predictive models to anticipate no-shows. We use locally collected dataset to develop the predictive models. The key feature utilized in the developing the predictive models is the patient prior no-show history. Thus, we propose a novel representation as a binary sequence of events that the model can effectively learn the behavior of the attendance. The results show that our approach is both viable and generalizable. The experiments show the best performing model achieves AUC of 0.718, and the developed models outperformed the baseline approach with a percentage increase of over 62%. Finally, we discuss various applications, such as a personalized recommender system for appointment time, that could be built on the deployed models to boost the utilization of the dental clinic resources and increase overall patient satisfaction. Our code and the dataset are freely available for the community to reproduce and investigate other ideas.

In future work, we plan to collect the dataset from the same dental clinics to study the patient no-show problem during and post the outbreak of the COVID-19 pandemic and how the results are different before the pandemic. Additionally, combining predictive no show model and cancellation model is another promising direction that may reduce the cost and utilize the healthcare clinic resources effectively.

## Supplemental Information

10.7717/peerj-cs.1147/supp-1 Supplemental Information 1 Dental appointments using to train and test machine learning models. Each line represents an appointment along with a set of variables

10.7717/peerj-cs.1147/supp-2 Supplemental Information 2 Jupyter notebook containing all steps to preprocess train and test the data for various experiments.

## Additional Information and Declarations

### Data Availability

The following information was supplied regarding data availability: The raw data is available in the Supplemental Files.
